# Effects of pullulan, beet, and *Artemisia princeps* coatings on quality of table eggs during room temperature storage

**DOI:** 10.1016/j.psj.2025.105653

**Published:** 2025-08-06

**Authors:** H.N. Lee, G.L. Yeom, Y.B. Kim, J.Y. Park, G.Y. Park, J.W. Shin, W. Park, J. Choi, J.H. Cho, J.H. Kim

**Affiliations:** Department of Animal Science, Chungbuk National University, Cheongju 28644, Republic of Korea

**Keywords:** *Artemisia princeps*, Beet, Egg coating, Egg quality, Pullulan

## Abstract

The objective of this experiment was to investigate effects of pullulan (PL), beet (BT), and *Artemisia princeps* (AP) coatings on quality of table eggs during room temperature storage. A total of 1,200 non-fertile eggs were collected from 48-wk-old Hy-Line Brown laying hens. These eggs were randomly allotted to 4 treatments with 6 replicates consisting of 10 eggs per replicate. A total of 60 eggs per treatment were analyzed weekly throughout the study. Uncoated eggs were used for the CON group, while eggs coated with PL, BT, and AP were used for PL, BT, and AP groups, respectively. Results indicated that BT and AP groups exhibited greater (*P* < 0.05) Haugh unit than CON and PL groups. Albumen height was greater (*P* < 0.05) in PL, BT, and AP groups than in the CON group. PL, BT, and AP groups had greater (*P* < 0.05) yolk height than the CON group. The yolk index was less (*P* < 0.05) in PL and CON groups than in BT and AP groups. Albumen and yolk pH were greater (*P* < 0.05) in PL, BT, and AP groups than in the CON group. The foam volume was increased (*P* < 0.05) in all coated groups compared to that in the CON group. PL and AP groups had greater (*P* < 0.05) total microbial counts than CON and BT groups. In conclusion, PL-based egg coatings, particularly those blended with AP, were effective in maintaining egg quality and egg freshness during room temperature storage.

## Introduction

Eggs are a rich source of protein and one of the most important components of human nutrition (Yüceer and Caner, 2014). However, immediately after being laid, eggs begin to deteriorate and their quality declines (Yüceer and Caner, 2014; Oliveira et al., 2020). Egg quality is influenced by various factors such as time, temperature, and storage period (Samli et al., 2005; Yüceer and Caner, 2014). Coating and refrigeration are effective methods for maintaining egg quality during short-term storage (Caner, 2005; Wardy et al., 2010; Homsaard et al., 2021; Al Awwaly et al., 2024). Currently, food packaging materials such as coatings and edible films are widely studied for their potential to extend shelf lives of food products (Morsy et al., 2015; Kraśniewska et al., 2019; Mendonça et al., 2024; Purnawarman et al., 2025). Food coatings are known to function as semi-permeable barriers against gases and moisture, thereby reducing respiration, water loss, and oxidative reactions (Caner, 2005). In addition, these coatings can seal pores of the eggshell, which can prevent penetration of microorganisms (Yüceer and Caner, 2014; Xu et al., 2018). This characteristic can enhance the shelf life and maintain the quality of eggs (Eddin and Tahergorabi, 2019).

Pullulan (PL) is an extracellular polysaccharide produced by *Aureobasidium pullulans*. It is water-soluble, odorless, tasteless, non-toxic, and biodegradable (Cheng et al., 2011; Kraśniewska et al., 2019). Moreover, it exhibits low oxygen permeability and favorable barrier properties (Singh et al., 2008). Therefore, PL-based coatings have been widely applied to preserve the quality of fresh products. Ganduri (2020) reported that pullulan-based edible active coating prolonged the shelf life of banana. In addition, Wani et al. (2024) demonstrated that PL-based coating extended the shelf-life and maintained the quality of cherries. However, PL lacks active functionalities such as antimicrobial and antioxidant activities (Tabasum et al., 2018; An et al., 2024). Morsy et al. (2015) have demonstrated that egg coatings formulated with PL, either alone or in combination with nisin have positive effects on egg quality. Similarly, Kumar et al. (2021) observed that a chitosan-pullulan composite coating incorporated with pomegranate peel extract positively affected the shelf life of mangoes. However, research on pullulan-based coating for eggs remains limited compared to studies on their application in fruit and vegetables.

Furthermore, recent studies have also investigated the use of natural compounds into various polysaccharide-based coatings for egg preservation. Song et al. (2022) demonstrated that an egg coating composed of chitosan-shellac combined with pine needle essential oil exhibited antibacterial effects and maintained egg quality. Similarly, Hejazian et al. (2023) reported that a chia seed mucilage-based coating with turmeric essential oil effectively preserved egg quality. Therefore, the functional properties of PL-based coatings may be enhanced by blending them with natural materials such as essential oils or plant extracts (Pan et al., 2014; Kraśniewska et al., 2019).

In response to growing consumer demand for safe and healthy food, artificial additives such as sweeteners, preservatives, and flavorings are increasingly avoided (Khalil et al., 2025). Accordingly, these additives have been replaced with natural alternatives (Echegaray et al., 2023). Beet (*Beta vulgaris*; BT) contains various bioactive compounds, including betalains, flavonoids, terpenoids, saponins, vitamins, phenolic acids, steroids, alkaloids, tannins, and sugars (Stoica et al., 2025). Especially, betalains in BT have high antioxidant capacity with a potential role in the prevention and treatment of various diseases (Chen et al., 2021; Nirmal et al., 2024). *Artemisia princeps* (AP) contains abundant phenolic and flavonoid compounds with notable bioactivities, including antioxidant and antimicrobial effects (Park et al., 2009; El Rabey and Almutairi, 2025). The AP contains various flavonoids, including eupatilin and jaceosidin (Chang et al., 2009), which have been reported to possess anti-inflammatory, anticancer, and antioxidant activity (Min et al., 2009; Lee et al., 2023). Based on these characteristics, BT and AP are commonly used as food additives and natural colorants (Park et al., 2009; Clifford et al., 2015).

Based on these characteristics, blending natural materials with PL coatings may enhance egg quality and improve antimicrobial activity. Therefore, the objective of this experiment was to investigate the effects of coating eggs with PL and PL blended with natural materials such as BT and AP on egg quality during room temperature storage.

## Materials and methods

A total of 1,200 non-fertile eggs were obtained from 48-wk-old Hy-Line Brown laying hens of a local commercial farm (Hansol Farm, Eumseong, Republic of Korea). To ensure freshness, the eggs were coated immediately after collection. All eggs were used randomly divided into the following 4 treatments with 6 replicates, each replicate consisting of 10 eggs in a completely randomized design: 1) uncoated eggs (CON group), 2) eggs coated with PL (PL group), 3) eggs coated with PL and BT (BT group), and 4) eggs coated with PL and AP (AP group). During the study period, 60 eggs from each treatment were analyzed.

### Coating solutions and coating of eggshell

Egg coating was prepared according to methods described by Eddin and Tahergorabi (2019) and Oliveira et al. (2020). Coating solutions were prepared using 50 g of PL powder (Hayashibara Co., Ltd., Okayama, Japan) and 1 L of distilled water. These solutions were mixed at 50°C for 20 min. After 5 g of glycerine (99 %; Samchun Chemicals, Seoul, Republic of Korea) was added, the mixture was mixed for 5 min. For preparing BT and AP solutions, PL solution was added with 50 g of BT powder (Charm goods, Seongnam, Republic of Korea) and 50 g of AP powder (Young miller, Gimhae, Republic of Korea), respectively. Eggs were individually submerged in the coating solutions for 30 s and dried at room temperature for 10 min. After drying, all eggs were stored at 25°C for 4 wk.

### Egg weight

Egg quality was analyzed at 0, 1, 2, 3, and 4 wk. Egg weight was measured using a digital egg tester (DET 6500, NABEL Co., Ltd., Kyoto, Japan). Egg weight loss was calculated based on the difference between the initial and final egg weights using the following formula: Egg weight loss (%) = [(initial egg weight – final egg weight) / initial egg weight] × 100. Egg yolk and eggshell weights were measured using an electronic auto balance (HS-1000A, Hansung Instrument, Gwangmyeong, Republic of Korea). Egg albumen weight was calculated by separating egg yolk and egg albumen and subtracting the egg yolk weight and eggshell weight from the total egg weight.

### Haugh unit and egg grade

Haugh unit (HU) was measured using a digital egg tester. HU values were calculated from egg weight (W) and egg albumen height (H), using the method described by Haugh (1937): HU= 100 log (H – 1.7 W^0.37^ + 7.6). Egg grade was determined according to USDA (2000) criteria: AA = HU above 72, *A* = HU between 71 and 60, and *B* = HU below 60. Egg albumen height was measured using a digital egg taster.

### Eggshell quality

Eggshell strength and eggshell thickness were measured using a digital egg taster.

### Eggshell and yolk color

Egg color was expressed as CIE Lab value of lightness (L*), redness (a*), and yellowness (b*). Eggshell color and egg yolk color were measured using a color reader (model CR-10, Konica Minolta Optics Inc., Tokyo, Japan).

### Egg yolk quality

Egg yolk score, egg yolk diameter, egg yolk index (YI), and egg yolk height were measured using a digital egg taster. YI values were calculated from egg yolk height and egg yolk diameter, using the method described by Sharp and Powell (1930): YI= egg yolk height (mm) / egg yolk diameter (mm). For measuring egg yolk moisture, a 1 g sample was added to a crucible and dried at 105°C for 24 h using a drying oven (HB-501 M, Hanbaek Scientific Co., Bucheon, Republic of Korea).

### Egg albumen and yolk pH

The pH was analyzed by separating egg yolk and albumen. Egg yolk and albumen pH were measured using a pH meter (HI99163, Hanna Instruments, Rhode Island).

### Foam volume of albumen and Thiobarbituric acid reactive substance (TBARS) of yolk

Foam volume was analyzed at 0, 2, and 4 wk. Foam volume was measured using a method described by Silversides and Budgell (2004). Egg albumen was obtained using an egg yolk separator. Approximately 60 g of albumen was mixed at 1 min and 30 s in a bowl using a hand mixer (OFM-309, Zhongshan Lvhang Electric Co., Ltd., Guangdong, China). Foam volume was calculated in milliliters of foam per gram of albumen.

Thiobarbituric acid reactive substance (TBARS) of egg yolk was measured at 0, 2, and 4 wk. The sample was prepared using 5 g of egg yolk, 50 µL of butylated hydroxytoluene, and 15 mL of distilled water. The sample was homogenized for 30 s. Then 1 mL of the mixture and 2 mL of TBA/trichloroacetic acid were added to a 15 mL tube (Conical tube 50015, SPL Life Science Co., Ltd., Pocheon, Republic of Korea). The mixture was heated at 90°C for 15 min using a water bath (MaXturdy^TM^45, Daihan Scientific Co., Ltd., Wonju, Republic of Korea). After heating, the mixture was cooled and then centrifuged at 3,000 rpm for 10 min. The supernatant was collected and absorbance at 531 nm was detected using a microplate reader (INNO Microplate Spectrophotometer, LTEK, Seongnam-si, Gyeonggi-do, Republic of Korea).

### Eggshell microbial counts

Microbial counts were measured at 0, 2, and 4 wk in accordance with the methodology reported by Kabploy et al. (2023). Approximately 25 g of eggshell and 225 mL of peptone solution (MB-B2220, KisanBio Co., Ltd., Seoul, Republic of Korea) were added to a sterile sample bag (3 M 1523FW Sample bag, 3 M Company, Saint Paul, MN). The sample bag was then placed in an incubator (MIR-262, SANYO Electric Co., Ltd., Osaka, Japan) at 37°C for 24 h. After incubation, the culture solution and phosphate-buffered saline (LB 204-02, Wel, WELGENE Inc., Gyeonsan, Republic of Korea) were diluted (1:10) and plated on agar. Total microbial counts were analyzed using a plate count agar (MB-P1040, KisanBio Co., Ltd., Seoul, Republic of Korea). *Staphylococcus* counts were measured using mannitol salt agar (MB-M1029, KisanBio Co., Ltd., Seoul, Republic of Korea) plates.

### Ultrastructure assessment

Ultrastructure assessment was analyzed at the end of the study. Before conducting ultrastructure assessment, eggshell membranes were removed using distilled water for each treatment. After removal, eggshells were dried at room temperature for 24 h. These dried eggshells were then sliced into 1 cm^2^ pieces and coated with gold-palladium (Q150T ES Plus, Quorum Technologies Ltd., Laughton, United Kingdom). Coated samples were observed using a field emission scanning electron microscope (SEM; GeminiSEM 560, Zeiss Co. Ltd., Oberkochen, Germany) at a standard magnifications of x 500.

### Statistical analysis

All data were analyzed using the PROC MIXED procedure of SAS (SAS Institute., Cary, NC) as a 1-way ANOVA in a completely randomized design. Each replicate was considered the experimental unit for all analyses. All data were checked for normal distribution and outliers with the UNIVARIATE procedure of SAS (Steel et al., 1997). The LSMEANS procedure was used to calculate mean values and the PDIFF option was used to separate the means if the difference was significant. Significance for statistical test was set at *P* < 0.05.

## Results and discussion

The initial eggs exhibited a HU value of 87.05, classifying them as AA-grade quality according to USDA standards (USDA, 2000). Other internal quality attributes also met the reference criteria established by national standards in Korea (MAFRA, 2025).

### Egg weight

Egg weight loss showed no significant differences among treatment groups (Table 1). Eggshell weight differed significantly (*P* < 0.05) among groups from 2 wk. The AP group had greater (*P* < 0.05) eggshell weight at 4 wk than the CON and PL groups. The CON and BT groups had greater (*P* < 0.05) albumen weight at 3 wk than the PL and AP groups. No significant differences were observed among groups during wk 1, 2, and 4. Egg yolk weight at 1 wk was less (*P* < 0.05) in the PL group than in CON and BT groups. The AP group had greater (*P* < 0.05) yolk weight at 3 wk than the CON group. However, egg yolk weight at 2 and 4 wk was not significantly different among treatment groups.

**Table 1:** Effects of eggs coated with pullulan, beet, and *Artemisia princeps* on egg weight^1^.

	Egg weight loss, %				
Coating^2^	0 wk	1 wk	2 wk	3 wk	4 wk
CON	0.00	1.12	2.74	3.67	4.75
PL	0.00	0.88	2.78	3.85	4.22
BT	0.00	0.40	2.21	2.94	3.86
AP	0.00	0.41	2.42	4.24	4.63
SEM		2.437	0.891	0.479	0.725
*P*-value		0.989	0.943	0.165	0.769

	Eggshell weight, g				
Coating^2^	0 wk	1 wk	2 wk	3 wk	4 wk
CON	8.5	8.3	8.1^b^	8.1^b^	8.0^c^
PL	8.3	8.3	8.4^a^	8.4^a^	8.1^b^^c^
BT	8.6	8.5	8.6^a^	8.3^a^^b^	8.3b^a^^b^
AP	8.5	8.5	8.6^a^	8.5^a^	8.5^a^
SEM	0.08	0.08	0.09	0.08	0.11
*P*-value	0.051	0.125	<0.001	0.021	0.007
	Albumen weight, g				
Coating^2^	0 wk	1 wk	2 wk	3 wk	4 wk

CON	38.6	37.4	36.3	36.1^a^	34.5
PL	38.0	37.1	36.7	35.6^a^^b^	34.0
BT	38.0	37.2	36.3	35.9^a^	33.7
AP	38.7	37.2	36.4	34.6^b^	33.5
SEM	0.33	0.34	0.33	0.37	0.43
*P*-value	0.237	0.929	0.790	0.030	0.414
	Egg yolk weight, g				
Coating^2^	0 wk	1 wk	2 wk	3 wk	4 wk

CON	16.5	17.7^a^	17.5	17.1^b^	18.2
PL	16.4	17.0^b^	17.6	17.5^a^^b^	18.4
BT	16.8	17.5^a^	17.4	17.5^a^^b^	18.9
AP	16.5	17.4^a^^b^	17.4	17.9^a^	18.7
SEM	0.13	0.16	0.18	0.19	0.26
*P*-value	0.166	0.016	0.841	0.025	0.155

a-c Means within a variable with no common superscript differ significantly (*P* < 0.05).

1 All means are average of 6 replicates per treatment.

2 CON = uncoated egg; PL = coated with pullulan; BT = coated with pullulan + beet powder; AP = coated with pullulan + *Artemisia princeps* powder.

Egg weight is an important factor that influences grading standards and the relative proportions of albumen, egg yolk, and eggshell (Farooq et al., 2001; Khurshid et al., 2003). In addition, Şekeroğlu and Altuntaş (2008) demonstrated that egg weight significantly affects yolk height, YI, and yolk color. Therefore, egg weight loss is considered a critical factor in determining egg quality (Xu et al., 2018). In the present study, egg coating did not significantly influence egg weight loss. This finding contrasts with the results of Oliveira et al. (2023), who reported that egg coating with green banana flour and Tahiti lemon essential oil significantly reduced egg weight loss compared to uncoated eggs. In addition, de Araújo et al. (2023) observed that quail eggs coated with a corn starch-based film combined with basil essential oil showed decreased egg weight loss. Similarly, Kumar et al. (2021) reported that coating mangoes with chitosan-pullulan and pomegranate peel extract reduced mango fruit weight loss. This result may be attributed to the protective role of the coating. Caner (2005) suggested that improved protective methods, such as eggshell coating, may help minimize moisture and weight losses during storage. Therefore, it may be inferred that egg coating did not exert any adverse effects on egg weight loss.

### Haugh unit and egg grade

The HU varied significantly (*P* < 0.05) among groups over the entire storage period (Table 2). The HU at 4 wk greater (*P* < 0.05) in the BT and AP groups than in the CON and PL groups. The CON group decreased from grade AA to grade A after 1 wk and to grade B after 3 wk. The egg grade of the PL group dropped from grade AA to grade A and then to grade B after 4 wk. Egg grade changed from grade AA to grade A after 3 wk for BT and AP groups.

**Table 2:** Effects of eggs coated with pullulan, beet, and *Artemisia princeps* on Haugh unit and egg grade^1^.

	Haugh unit and egg grade^3^				
Coating^2^	0 wk	1 wk	2 wk	3 wk	4 wk
CON	89.1^a^ (AA)	68.3^d^ (A)	61.0^c^ (A)	52.9^c^ (B)	53.9^c^ (B)
PL	87.6^a^^b^ (AA)	71.9^c^ (A)	70.3^b^ (A)	60.8^b^ (A)	58.4^b^ (B)
BT	86.3^b^^c^ (AA)	79.8^a^ (AA)	75.3^a^ (AA)	68.6^a^ (A)	66.1^a^ (A)
AP	85.2^c^ (AA)	76.7^b^ (AA)	73.1^ab^ (AA)	69.3^a^ (A)	67.9^a^ (A)
SEM	0.75	0.93	1.11	1.18	1.37
*P*-value	0.002	0.002	<0.001	<0.001	<0.001

	Albumen height, mm				
Coating^2^	0 wk	1 wk	2 wk	3 wk	4 wk
CON	8.2^a^	5.2^d^	4.4^c^	3.7^c^	3.7^c^
PL	7.8^ab^	5.3^c^	5.5^b^	4.4^b^	4.1^b^
BT	7.7^b^	6.7^a^	6.0^a^	5.2^a^	4.9^a^
AP	7.5^b^	6.2^b^	5.7^ab^	5.2^a^	5.1^a^
SEM	0.13	0.13	0.13	0.12	0.14
*P*-value	0.002	<0.001	<0.001	<0.001	<0.001

^a-d^Means within a variable with no common superscript differ significantly (*P* < 0.05).

1 All means are average of 6 replicates per treatment.

2 CON = uncoated egg; PL = coated with pullulan; BT = coated with pullulan + beet powder; AP = coated with pullulan + *Artemisia princeps* powder.

3 Egg grade: AA = HU > 72; *A* = 71 to 60; *B* = HU < 60 (USDA, 2000).

Significant differences (*P* < 0.05) in egg albumen height were observed among treatment groups throughout all storage periods. Egg albumen height at 4 wk was less (*P* < 0.05) in the CON group than in the PL, BT, and AP groups.

Our findings also confirmed that egg coating contributed to the maintenance of HU, an important indicator of internal egg quality. This observation was consistent with the findings of Shurmasti et al. (2023), who reported that coating eggs with polyvinyl alcohol/chitosan helped maintain HU values. Similarly, Eddin and Tahergorabi (2019) observed that egg coating using sweet potato and essential oils has beneficial effects on HU values. Caner and Yüceer (2015) suggested that uncoated eggs exhibit less HU values than those coated with protein–based materials. This improvement in HU values is not due to a direct effect of the coating, but rather to its role in preserving albumen integrity and slowing the decline in HU over time (Pires et al., 2021). In our study, PL-based egg coating effectively preserved albumen quality. Weakening of albumen over time occurs due to biochemical reactions, such as ovomucin proteolysis (Chen et al., 2005; Yüceer and Caner, 2014). Ovomucin is a sulphated glycoprotein in egg albumen. It is characterized by a high molecular weight with a subunit structure (Hiidenhovi, 2007). Proteolytic degradation of ovomucin transforms thick albumen into a thin albumen, leading to reductions of albumen height and HU values (Robinson and Monsey, 1971; Alleoni, 2006; Oliveira et al., 2022). Therefore, egg coating may have delayed ovomucin proteolysis, thereby contributing to the maintenance of greater HU values.

Eggs are classified into three quality grades based on their HU: “AA” indicating an HU above 72, “A” representing an HU between 71 and 61, and “B” indicating an HU below 60 (USDA, 2000). In the CON group, egg grade was decreased from grade AA to A after 1 wk and further decreased to grade B after 3 wk. In the PL group, egg graded dropped from grade AA to A after 1 wk and to grade B after 4 wk. In contrast, egg grade was maintained at AA for 3 wk in BT and AP groups and then decreased to grade A after 4 wk. In another study, eggs coated with PL and PL containing nisin showed a gradual reduction of grade from AA to A to B after 6 wk, whereas uncoated eggs exhibited more rapid quality degradation (Morsy et al., 2015). Furthermore, Yüceer and Caner (2014) demonstrated that eggs coated with lysozyme–chitosan and chitosan maintained higher grades than uncoated eggs. This difference is attributed to variations in albumen quality among treatment groups. Egg grade is primarily determined by egg weight and albumen quality (Stadelman, 1986; Morsy et al., 2015). In our study, PL, BT, and AP groups showed greater albumen height than the CON group. Therefore, it may be concluded that egg coating contributes to egg grade maintenance by preserving albumen quality.

### Eggshell quality

Eggshell thickness at 3 wk was greater (*P* < 0.05) in the BT group than in CON, PL, and AP groups (Table 3). However, eggshell thickness at 1, 2, and 4 wk was not significantly different among treatment groups. Eggshell strength showed no significant differences among groups.

**Table 3:** Effects of eggs coated with pullulan, beet, and *Artemisia princeps* on eggshell quality^1^.

	Eggshell strength, kgf				
Coating^2^	0 wk	1 wk	2 wk	3 wk	4 wk
CON	4.7	4.6	4.7	4.8	4.5
PL	4.8	4.6	4.9	4.8	4.9
BT	4.6	4.8	5.0	4.9	4.7
AP	4.6	4.9	4.9	4.6	4.8
SEM	0.11	0.13	0.11	0.11	0.22
*P*-value	0.734	0.431	0.366	0.231	0.538

	Eggshell thickness, μm				
Coating^2^	0 wk	1 wk	2 wk	3 wk	4 wk
CON	411	420	412	412^b^	405
PL	421	418	412	405^b^	414
BT	416	418	410	423^a^	413
AP	417	421	410	411^b^	404
SEM	3.0	3.1	3.2	3.4	5.0
*P*-value	0.113	0.879	0.916	0.003	0.306

a,b Means within a variable with no common superscript differ significantly (*P* < 0.05).

1 All means are average of 6 replicates per treatment.

2 CON = uncoated egg; PL = coated with pullulan; BT = coated with pullulan + beet powder; AP = coated with pullulan + *Artemisia princeps* powder.

The eggshell serves as a protective barrier against various external forces, including mechanical impacts and damage during transport (Caner and Yüccer, 2015). In addition, pores in the eggshell facilitate fluid and gas exchange and serve as a barrier to microbial contamination (Caner and Yüccer, 2015). Therefore, eggshell integrity is considered a crucial economic factor in egg production (Biladeau and Kenner, 2009). In our study, egg coating had no significant effect on eggshell strength after 4 wk of storage. This result was consistent with findings of Pires et al. (2019), who reported that coated eggs with mineral oil and rice protein did not affect eggshell strength. In contrast, other studies have shown that coatings with protein-based or waxes exhibit enhanced eggshell strength compared to uncoated eggs (Biladeau and Keener, 2009; Caner and Yüccer, 2015). The reason for this result may be associated with physicochemical properties of the coating materials. Several previous studies have suggested that egg coatings may increase eggshell thickness, thereby improving eggshell strength (Canner and Yüccer, 2015; Pires et al., 2019). However, in our study, PL-based egg coating had no effect on eggshell thickness at 4 wk. Therefore, these findings suggest that PL-based coating may not be effective in enhancing eggshell strength.

### Eggshell color

Significant differences (*P* < 0.05) in eggshell color were observed among treatment groups throughout all storage periods (Table 4). L*, a*, and b* values at 4 wk were less (*P* < 0.05) in the AP group than in the CON, PL, and BT groups.

**Table 4:** Effects of eggs coated with pullulan, beet, and *Artemisia princeps* on eggshell color^1^.

	Eggshell color, L*				
Coating^2^	0 wk	1 wk	2 wk	3 wk	4 wk
CON	57.1^a^	56.5^b^	57.5^a^	58.6^a^	58.3^a^
PL	56.4^a^	60.3^a^	56.6^a^	57.3^b^	57.7^a^
BT	54.0^b^	56.1^b^	55.6^b^	55.5^c^	55.7^b^
AP	52.6^c^	52.5^c^	50.3^c^	51.7^d^	53.5^c^
SEM	0.39	0.46	0.34	0.36	0.44
*P*-value	<0.001	<0.001	<0.001	<0.001	<0.001

	Eggshell color, a*				
Coating^2^	0 wk	1 wk	2 wk	3 wk	4 wk
CON	21.2^a^	21.5^a^	24.0^a^	22.0^b^	23.6^a^
PL	20.7^a^	18.8^b^	24.1^a^	22.2^ab^	23.0^ab^
BT	20.7^a^	19.4^b^	23.2^b^	22.7^a^	22.7^b^
AP	14.1^b^	13.1^c^	17.1^c^	16.4^c^	15.9^c^
SEM	0.22	0.24	0.18	0.20	0.23
*P*-value	<0.001	<0.001	<0.001	<0.001	<0.001

	Eggshell color, b*				
Coating^2^	0 wk	1 wk	2 wk	3 wk	4 wk
CON	29.4^a^	28.8^a^	27.8^a^	26.3^a^	28.0^a^
PL	27.8^b^	27.2^c^	26.5^b^	25.4^b^	26.7^b^
BT	25.6^c^	27.9^b^	26.4^b^	25.5^b^	26.4^b^
AP	25.1^d^	24.5^d^	23.5^c^	22.6^c^	23.2^c^
SEM	0.16	0.17	0.17	0.19	0.27
*P*-value	<0.001	<0.001	<0.001	<0.001	<0.001

a-d Means within a variable with no common superscript differ significantly (*P* < 0.05).

1 All means are average of 6 replicates per treatment.

2 CON = uncoated egg; PL = coated with pullulan; BT = coated with pullulan + beet powder; AP = coated with pullulan + *Artemisia princeps* powder.

Eggshell color is one of the important factors influencing consumer purchasing decisions (Caner and Cansiz, 2008; Eddin and Tahergorabi, 2019). In the present study, BT and AP groups exhibited different L*, a*, and b* values of eggshell color compared to the CON group. The reason for this result may be associated with natural pigments present in BT and AP. Red beetroot is commonly used as a natural colorant and food additive in the food industry (Slavov et al., 2013; Chhikara et al., 2019). In addition, AP contains green pigments such as chlorophyll (Park, 2020). Therefore, the pigments derived from BT and AP may have contributed to the observed difference in eggshell color.

### Egg yolk color

The egg yolk color varied significantly (*P* < 0.05) among groups over the entire storage except for 2 wk (Table 5). The CON group had less (*P* < 0.05) L* value at 4 wk than the other groups. The PL group had greater (*P* < 0.05) b* value at 4 wk than the CON, BT, and AP groups.

**Table 5:** Effects of eggs coated with pullulan, beet, and *Artemisia princeps* on egg yolk color^1^.

	Egg yolk color, L*				
Coating^2^	0 wk	1 wk	2 wk	3 wk	4 wk
CON	61.1^a^	61.1^a^	63.1	62.1	58.5^c^
PL	58.0^b^	58.0^b^	63.9	62.5	61.4^a^
BT	61.0^a^	61.0^a^	62.7	61.8	60.2^b^
AP	59.1^b^	59.1^b^	62.8	61.6	60.7^ab^
SEM	0.59	0.59	0.34	0.38	0.41
*P*-value	<0.001	<0.001	0.070	0.375	<0.001

	Egg yolk color, a*				
Coating^2^	0 wk	1 wk	2 wk	3 wk	4 wk
CON	10.1^a^	10.5	16.0	16.6^a^	16.4
PL	9.0^b^	10.7	16.1	15.9^b^	16.5
BT	10.0^a^	10.9	15.5	15.5^b^	16.0
AP	9.2^b^	10.5	15.6	15.6^b^	16.2
SEM	0.21	0.19	0.21	0.19	0.21
*P*-value	<0.001	0.586	0.162	<0.001	0.340

	Egg yolk color, b*				
Coating^2^	0 wk	1 wk	2 wk	3 wk	4 wk
CON	51.0^a^	52.3	53.6	58.1^a^	53.9^b^
PL	46.1^b^	51.1	54.2	55.4^b^	57.0^a^
BT	50.6^a^	52.1	52.2	53.1^c^	53.8^b^
AP	46.8^b^	51.8	52.6	53.5^c^	54.4^b^
SEM	0.66	0.51	0.63	0.56	0.66
*P*-value	<0.001	0.409	0.102	<0.001	0.002

a-c Means within a variable with no common superscript differ significantly (*P* < 0.05).

1 All means are average of 6 replicates per treatment.

2 CON = uncoated egg; PL = coated with pullulan; BT = coated with pullulan + beet powder; AP = coated with pullulan + *Artemisia princeps* powder.

In our study, L* and b* values of egg yolk color were significantly different among groups. This result was similar to findings of Caner (2005), who reported that eggs coated with whey protein isolate showed differences in egg yolk color of a* value among groups. However, Caner and Cansiz (2008) observed no significant differences in yolk color for eggs coated with chitosan containing organic acid. Similarly, eggs coated with cassava starch biopolymer enriched with different essential oils did not show significant differences in yolk color (Oliveira et al., 2022). These variations may be attributed to the presence of yolk carotenoids. Egg yolk color is primarily determined by carotenoids such as lutein and cryptoxanthin (Caner, 2005). Carotenoids can be degraded by oxidative processes, which may lead to changing the yolk pigmentation during storage (Caner, 2005). Kojima et al. (2022) demonstrated that dietary supplementation of carotenoids in laying hens led to varying degrees of carotenoid accumulation in egg yolks depending on the breed. Therefore, egg yolk color may be partially attributed to the applied coating, but it may also be influenced by breed and the level of carotenoid accumulation (Caner and Cansiz, 2008).

### Egg yolk quality

Egg yolk color fan score differed significantly (*P* < 0.05) among treatment groups from 3 wk (Table 6). The CON group had greater (*P* < 0.05) egg yolk color at 4 wk than the BT and AP groups. Significant differences (*P* < 0.05) in egg yolk diameter were observed among groups at all storage periods. The BT group had less (*P* < 0.05) yolk diameter at 4 wk than the CON, PL, and AP groups. The YI value varied significant (*P* < 0.05) among treatment groups during all storage periods. The AP group had greater (*P* < 0.05) YI at 4 wk than the CON and PL groups. Egg yolk height was greater (*P* < 0.05) in the BT and AP groups than in the CON and PL groups throughout the 4-wk storage period. The egg yolk moisture at 1 wk was greater (*P* < 0.05) in the CON group than in PL, BT, and AP groups. The AP group had less (*P* < 0.05) yolk moisture at 2 wk than CON and PL groups. The CON group had greater (*P* < 0.05) yolk moisture at 3 wk than PL and BT groups. However, egg yolk moisture at 4 wk showed no significant differences among groups.

**Table 6:** Effects of eggs coated with pullulan, beet, and *Artemisia princeps* on egg yolk quality^1^.

	Egg yolk color fan				
Coating^2^	0 wk	1 wk	2 wk	3 wk	4 wk
CON	6.7^a^	6.8	6.9	7.6^a^	8.0^a^
PL	6.5^ab^	6.9	7.0	7.2^b^	7.7^ab^
BT	6.7^a^	6.9	6.9	7.3^b^	7.3^b^
AP	6.4^b^	6.7	6.8	7.3^b^	7.4^b^
SEM	0.08	0.07	0.08	0.09	0.12
*P*-value	0.024	0.093	0.473	0.010	<0.001

	Eggs yolk diameter, mm				
Coating^2^	0 wk	1 wk	2 wk	3 wk	4 wk
CON	40.9	40.6^b^	45.1^a^	46.8^a^	47.4^a^
PL	40.2	40.2^b^	43.8^b^	45.0^b^	45.2^ab^
BT	40.0	41.8^a^	42.8^c^	44.2^c^	39.8^c^
AP	40.5	40.6^b^	42.9^c^	43.7^c^	43.1^b^
SEM	0.38	0.32	0.27	0.30	0.83
*P*-value	0.386	0.005	<0.001	<0.001	<0.001

	Egg yolk index				
Coating^2^	0 wk	1 wk	2 wk	3 wk	4 wk
CON	0.43	0.38^b^	0.30^c^	0.24^c^	0.24^c^
PL	0.45	0.39^b^	0.34^b^	0.30^b^	0.28^bc^
BT	0.43	0.41^a^	0.37^a^	0.34^a^	0.37^ab^
AP	0.43	0.42^a^	0.37^a^	0.34^a^	0.41^a^
SEM	0.005	0.004	0.004	0.005	0.033
*P*-value	0.113	<0.001	<0.001	<0.001	0.001

	Egg yolk height, mm				
Coating^2^	0 wk	1 wk	2 wk	3 wk	4 wk
CON	17.6^ab^	15.4^b^	13.4^c^	11.4^c^	11.3^c^
PL	17.8^a^	15.6^b^	14.8^b^	13.6^b^	12.7^b^
BT	17.3^b^	17.0^a^	15.8^a^	14.9^a^	14.7^a^
AP	17.3^b^	16.8^a^	15.8^a^	14.6^a^	14.6^a^
SEM	0.12	0.14	0.13	0.15	0.18
*P*-value	0.004	<0.001	<0.001	<0.001	<0.001

	Egg yolk moisture, %				
Coating^2^	0 wk	1 wk	2 wk	3 wk	4 wk
CON	50.0	50.5^a^	51.7^a^	52.1^a^	52.1
PL	49.2	49.3^b^	51.0^ab^	50.9^b^	52.3
BT	49.5	49.1^b^	50.4^b^^c^	50.4^b^	53.0
AP	48.2	49.1^b^	49.9^c^	51.2^ab^	51.5
SEM	0.32	0.34	0.26	0.35	0.47
*P*-value	0.080	0.025	<0.001	0.017	0.178

a-c Means within a variable with no common superscript differ significantly (*P* < 0.05).

1 All means are average of 6 replicates per treatment.

2 CON = uncoated egg; PL = coated with pullulan; BT = coated with pullulan + beet powder; AP = coated with pullulan + *Artemisia princeps* powder.

The YI is calculated based on yolk height and yolk diameter (Yüceer and Caner, 2014). In the present study, the AP group exhibited greater YI at 4 wk than CON and PL groups. Jariyapamornkoon et al. (2024) demonstrated that eggs coated with durian rind pectin combined with nisin had greater YI value than uncoated eggs. Similarly, Oliveira et al. (2025) reported that that uncoated eggs had less YI value than coated quail eggs with corn starch and green propolis extract. These results were associated with differences in albumen quality. Oliveira et al. (2025) noted that the decline in yolk quality is closely linked to the deterioration of albumen quality. The release of CO_2_ and H_2_O from the egg interior into the external environment increases the albumen pH by disrupting the carbonic acid (H_2_CO_3_) buffering system, resulting in increased albumen fluidity (Cedro et al., 2009). The thinner albumen can migrate through the vitelline membrane into the yolk, negatively affecting yolk quality such as yolk diameter and height (Obanu and Mpieri, 1984; Watkins, 2007; Eke et al., 2013). Therefore, PL-based coatings help preserve yolk quality by maintaining higher YI values during storage.

### Egg pH

Egg albumen pH values at 1, 2, 3, and 4 wk were greater (*P* < 0.05) in the CON group than in the PL, BT, and AP groups (Table 7). The CON group had greater (*P* < 0.05) yolk pH values at 3 and 4 wk than the PL, BT, and AP groups. However, egg yolk pH values at 1 and 2 wk were not significantly different among groups.

**Table 7:** Effects of eggs coated with pullulan, beet, and *Artemisia princeps* on egg pH^1^.

	Albumen pH				
Coating^2^	0 wk	1 wk	2 wk	3 wk	4 wk
CON	8.6^a^	9.2^a^	9.3^a^	9.3^a^	9.3^a^
PL	8.5^b^	8.9^b^	9.0^b^	9.1^b^	9.1^b^
BT	8.5^b^	8.5^c^	8.6^d^	9.1^b^	8.7^c^
AP	8.5^b^	8.5^c^	8.7^c^	8.8^c^	8.7^c^
SEM	0.02	0.02	0.02	0.02	0.03
*P*-value	0.002	<0.001	<0.001	<0.001	<0.001

	Egg yolk pH				
Coating^2^	0 wk	1 wk	2 wk	3 wk	4 wk
CON	5.9	5.9	6.1	6.2^a^	6.4^a^
PL	5.9	5.9	6.1	6.1^b^	6.2^b^
BT	5.9	5.9	6.0	6.1^b^	6.2^b^
AP	5.8	6.0	6.0	6.1^b^	6.2^b^
SEM	0.05	0.05	0.03	0.02	0.04
*P*-value	0.258	0.491	0.503	<0.001	<0.001

a-d Means within a variable with no common superscript differ significantly (*P* < 0.05).

1 All means are average of 6 replicates per treatment.

2 CON = uncoated egg; PL = coated with pullulan; BT = coated with pullulan + beet powder; AP = coated with pullulan + *Artemisia princeps* powder.

Albumen pH is a widely used indicator of egg freshness (Pires et al., 2020). The pH of fresh albumen typically ranges from 7.5 to 8.5 (Waimaleongora-Ek et al., 2009). After prolonged storage, pH levels of both albumen and yolk are known to increase (Lee et al., 2016). In the present study, the CON group showed greater albumen and yolk pH at 4 wk than coated groups. Yüceer and Caner (2014) reported that eggs coated with chitosan and lysozyme have less albumen pH at 5 wk than uncoated eggs. Similarly, Eddin and Tahergorabi (2019) observed that sweet potato starch-based coated eggs have lower albumen pH at 5 wk than uncoated eggs. Mendonça et al. (2024) also reported that eggs coated with babassu starch and babassu oil exhibit lower yolk pH than uncoated eggs. These changes in pH are closely related to carbon dioxide loss from eggs. During storage, carbon dioxide escapes through eggshell pores (Kemps et al., 2007), leading to an increase in pH, a decrease in albumen height, and liquefaction of albumen (Waimaleongora-Ek et al., 2009). The liquefied albumen may subsequently migrate to egg yolk, altering the pH of both components (Pires et al., 2020). Previous studies have shown that egg coatings with low permeability may reduce carbon dioxide loss (Knight et al., 1972). Therefore, PL-based coating helps maintain lower pH levels in albumen and yolk, which contributes to improved internal egg quality during storage.

### Foam volume and thiobarbituric acid reactive substance (TBARS)

Egg foam volume at 2 wk was less (*P* < 0.05) in the CON group than in the PL, BT, and AP groups (Table 8). The CON group had greater (*P* < 0.05) foam volume at 4 wk than the PL, BT, and AP groups. TBARS of egg yolk showed no significant differences among groups (Table 9).

**Table 8:** Effects of eggs coated with pullulan, beet, and *Artemisia princeps* on foam volume^1^.

	Foam volume		
Coating^2^	0 wk	2 wk	4 wk
CON	4.3	3.3^b^	5.6^a^
PL	4.4	3.9^a^	4.9^b^
BT	4.2	4.1^a^	5.0^b^
AP	4.6	4.1^a^	4.8^b^
SEM	0.16	0.13	0.14
*P*-value	0.355	<0.001	0.003

a,b Means within a variable with no common superscript differ significantly (*P* < 0.05).

1 All means are average of 6 replicates per treatment.

2 CON = uncoated egg; PL = coated with pullulan; BT = coated with pullulan + beet powder; AP = coated with pullulan + *Artemisia princeps* powder.

**Table 9:** Effects of eggs coated with pullulan, beet, and *Artemisia princeps* on thiobarbituric acid reactive substance (TBARS)^1^.

	TBARS		
Coating^2^	0 wk	2 wk	4 wk
CON	0.65^b^	0.62	0.58
PL	0.83^a^	0.65	0.57
BT	0.66^b^	0.68	0.55
AP	0.60^b^	0.57	0.56
SEM	0.048	0.034	0.029
*P*-value	0.020	0.136	0.876

a,b Means within a variable with no common superscript differ significantly (*P* < 0.05).

1 All means are average of 6 replicates per treatment.

2 CON = uncoated egg; PL = coated with pullulan; BT = coated with pullulan + beet powder; AP = coated with pullulan + *Artemisia princeps* powder.

Egg foam volume is an important functional property in the production of manufactured egg products (Biladeau and Keener, 2009). In our study, coated groups had lower foam volumes at 4 wk than the uncoated group. This result was similar to findings of Biladeau and Keener (2009) who reported that eggs coated with oil, wax, whey protein isolate, or soy protein isolate exhibited less egg foam volumes at 12 wk than uncoated eggs. These results likely reflect variations in albumen pH. Egg coating helps prevent CO_2_ loss through the eggshell, thereby minimizing changes in albumen pH (Caner and Yüceer, 2015). Uncoated eggs show increased albumen pH during storage. This enhances protein unfolding and surface activity, leading to greater foam volume (Henry and Barbour, 1993). In our study, the CON group showed greater albumen pH than the PL, BT, and AP groups. Therefore, egg coating helps stabilize albumen pH and reduce foam volume, thereby preserving the functional properties of eggs during storage.

### Microbial counts

The AP group had less (*P* < 0.05) total microbial counts at 4 wk than the CON, PL, and BT groups (Table 10). However, total microbial counts at 2 wk were not significantly different among groups. The BT group had greater (*P* < 0.05) *Staphylococcus* counts at 2 wk than the CON, PL, and AP groups. However, *Staphylococcus* counts at 4 wk were not significantly different among groups.

**Table 10:** Effects of eggs coated with pullulan, beet, and *Artemisia princeps* on eggshell microbial counts^1^.

	Total microbial counts, log _10_ CFU/mL		
Coating^2^	0 wk	2 wk	4 wk
CON	6.5^a^	10.7	14.3^a^
PL	5.5^b^	10.8	13.6^b^
BT	6.4^a^	10.7	13.8^ab^
AP	5.3^b^	10.4	13.0^c^
SEM	0.11	0.18	0.13
*P*-value	0.002	0.598	0.011

	*Staphylococcus* counts, log _10_ CFU/mL		
Coating^2^	0 wk	2 wk	4 wk
CON	7.7^a^	7.4^b^	13.7
PL	6.8^b^	7.5^b^	13.6
BT	7.4^a^	9.0^a^	13.8
AP	7.6^a^	7.0^b^	13.0
SEM	0.11	0.18	0.48
*P*-value	0.011	0.005	0.562

a-c Means within a variable with no common superscript differ significantly (*P* < 0.05).

1 All means are average of 6 replicates per treatment.

2 CON = uncoated egg; PL = coated with pullulan; BT = coated with pullulan + beet powder; AP = coated with pullulan + *Artemisia princeps* powder.

The eggshell can be contaminated as the egg passes through the cloaca (Pires et al., 2022; Bermudez-Aguirre et al., 2025; Gao et al., 2025). In addition, contamination can occur due to improper storage and poor sanitation practices (Rachtanapun et al., 2022; Sabbah et al., 2024; Yu et al., 2025). The eggshell surface is primarily colonized by bacteria, including *Staphylococcus aureus* that can produce exotoxins, which can cause food poisoning (Ercoli et al., 2017; Pondit et al., 2018). In the current study, the PL and AP groups at 4 wk had less total microbial counts than the CON group. This result was similar to findings of a previous study of Oliveira et al. (2022), who observed that eggs coated with cassava starch biopolymer or essential oils showed lower total aerobic mesophilic bacteria counts than uncoated eggs. Similarly, Eddin and Tahergorabi (2019) reported that eggs coated with sweet potato starch-based coating containing thyme essential oil show significantly reduced *Salmonella enterica.* This antimicrobial effect may be associated with properties of coating materials. The PL has been reported to inhibit the growth of molds, yeasts, and bacteria by limiting O_2_ and CO_2_ exchange due to excellent gas-barrier properties (Chlebowska-Śmigiel and Gniewosz, 2009). The AP is known to possess antimalarial, antiviral, antioxidant, and anticancer activities (Tan et al., 1998). Alirezalu et al. (2022) reported that coating chicken breast meat with calcium-alginate containing AP showed greater inhibition of total viable counts, coliforms, molds, and yeasts. Therefore, PL and AP appear to contribute to the reduction of microbial load on eggshells through their antimicrobial activities.

However, in our study, the BT group exhibited greater *Staphylococcus* counts at 2 wk than other groups. This result may be associated with the composition of BT. The BT is known to be rich in sugar, with sucrose being the predominant sugar (Bavec et al., 2010; Hole et al., 2021). Dursun et al. (2022) reported that supplementation of glucose and sucrose increased *Staphylococcus* biofilm formation in culture media. Thus, the high sugar concentration in BT may have contributed to the increase of *Staphylococcus* counts observed in the BT group. However, this increase was not sustained, as no significant differences in *Staphylococcus* counts were observed at 3 and 4 wk. These findings suggest that the influence of high sugar content in BT on microbial growth may be transient (Robles-Gancedo et al., 2009; Kohout et al., 2020).

### Ultrastructure assessment

**Fig. 1:**
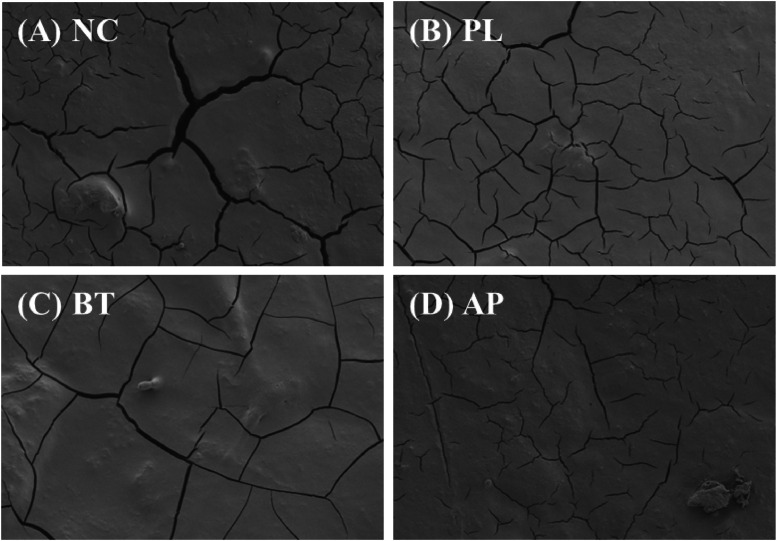
Effects of eggs coated with pullulan, beet, and *Artemisia princeps* on eggshell ultrastructure assessment (SEM x 500). (A) NC: uncoated eggs; (B) PL: egg coated with pullulan; (C) BT: egg coated with pullulan + beet powder; (D) AP: egg coated with pullulan + *Artemisia princeps* powder. The presence of deep fissures on the eggshell indicates patch cuticle coverage.

The SEM results of eggshell surface are presented in Fig. 1. PL-based egg coatings appeared to protect the cuticle and reduce deep fissures.

## Conclusions

In conclusion, PL-based egg coatings are effective in preserving egg quality during storage. Both BT and AP contribute to extending egg freshness. In particular, PL coating blended with AP exhibits antimicrobial effects. This study demonstrates that PL-based egg coatings blending natural materials may improve the shelf life of eggs. Thus, PL coatings blended with AP may help maintain egg quality and freshness. Furthermore, PL-based egg coatings can be effective in extending product shelf life and minimizing quality deterioration during storage. Therefore, these coatings offer economic potential for applications in the egg and food industries.

## CRediT authorship contribution statement

**H.N. Lee:** Conceptualization, Data curation, Formal analysis, Investigation, Methodology, Software, Writing – original draft, Writing – review & editing. **G.L. Yeom:** Formal analysis, Investigation, Methodology, Software, Writing – review & editing. **Y.B. Kim:** Formal analysis, Investigation, Methodology, Writing – review & editing. **J.Y. Park:** Formal analysis, Investigation, Methodology, Writing – review & editing. **G.Y. Park:** Formal analysis, Investigation, Methodology, Writing – review & editing. **J.W. Shin:** Formal analysis, Investigation, Methodology, Writing – review & editing. **W. Park:** Data curation, Methodology, Resources, Writing – review & editing. **J. Choi:** Data curation, Investigation, Methodology, Resources, Writing – review & editing. **J.H. Cho:** Conceptualization, Data curation, Methodology, Writing – review & editing. **J.H. Kim:** Conceptualization, Data curation, Funding acquisition, Methodology, Project administration, Supervision, Validation, Writing – original draft, Writing – review & editing.

## Disclosures

The authors declare that they have no known competing financial interests or personal relationships that could have appeared to influence the work reported in the present study.
